# Properties Enhancement of Oil Palm Trunk Plywood against Decay and Termite for Marine Applications

**DOI:** 10.3390/polym14132680

**Published:** 2022-06-30

**Authors:** Atikah Che Ismail, Sabiha Salim, Paridah Md Tahir, Seng Hua Lee, Muhammad Aizat Abd Ghani, Syeed SaifulAzry Al Edrus, Fadhlin Qayyum Ahmad Faisal

**Affiliations:** 1 Faculty of Forestry and Environment, Universiti Putra Malaysia, Serdang 43400, Selangor, Malaysia; atikahcheismail96@gmail.com; 2 Institute of Tropical Forestry and Forest Products, Universiti Putra Malaysia, Serdang 43400, Selangor, Malaysia; muhammad.aizat@upm.edu.my (M.A.A.G.); saifulazry@upm.edu.my (S.S.A.E.); fadhlinqayyum@gmail.com (F.Q.A.F.)

**Keywords:** marine plywood, oil palm trunk, veneer, phenolic resin, decay, termite

## Abstract

Oil palm trunk (OPT) veneers have the potential to be used in the production of plywood for marine applications. However, OPT is not resistant to fungal decay and termites, limiting its use in the production of marine plywood. As a result, in this study, phenolic resin treatment was used to improve the biological durability of OPT and produce marine grade equivalent (MGE) plywood. The OPT veneer was treated with medium molecular weight phenol formaldehyde (MmwPF) resin. The results showed that MmwPF resin with a solid content of 30% resulted in higher weight percent gain and polymer retention. Veneers treated with 30% MmwPF resin were then pressed for more than 10 min at temperatures above 140 °C. Dimensional stability, shear strength, bending strength, fungal decay resistance, and termite resistance were all tested on the plywood produced. The results of this study revealed that MGE plywood has satisfactory bonding quality and excellent biological durability. Good bending strength was recorded for the MGE plywood with modulus of rupture and modulus of elasticity ranged between 31.03 and 38.85 MPa and 4110 and 5120 MPa, respectively. Rubberwood, as a reference sample in this study, is not durable (Class 5) against white rot fungi and is moderately durable (Class III) against subterranean termite attacks. Interestingly, MGE plywood produced in this study was found very durable (Class 1) against white rot fungi. It is also durable (Class II) and very durable (Class I) against termite attacks, depending on the pressing parameters employed. Based on their outstanding bonding quality, bending strength, and biological durability, the study confirmed the feasibility of OPT plywood for marine applications.

## 1. Introduction

Marine plywood, as its name implies, is a type of plywood designated to stand up to the harsh marine environment. According to the definitions provided by BS 1088-1 [1], marine plywood is veneer plywood that has excellent resistance against fungal decay and superior bonding quality, enabling it suitable to be used for marine craft construction. The adhesive used for bonding the plies shall be a phenolic resin. In the case of using melamine–formaldehyde resin, it is essential to make sure that the resin contains sufficient resorcinol or other phenol to ensure a satisfactory bonding quality is obtained. Formaldehyde-based resins such as urea–formaldehyde (UF) and phenol–formaldehyde (PF) resins are the most prevalently used adhesive in bonding plywood [2,3,4], although some other adhesives are also being used [5,6,7]. However, UF resin is very susceptible to moisture and tends to emit high formaldehyde in high relative humidity and elevated temperature states. It is one of the main sources of indoor pollutants [8,9,10]. PF resin, on the other hand, is dimensionally stable and has excellent resistance to chemicals and heat [11]. Therefore, as stated in BS 1088-1 [1], phenolic resin is recommended as a binder for bonding plywood. Apart from that, the selection of timber species is crucial for the production of marine plywood. For standard marine plywood manufacturing, timbers with a density of more than 500 kg/m^3^ and a durability rating of Class 3 and above shall be used. As for lightweight marine plywood, timber species with a density lower than 500 kg/m^3^ and a durability rating of Class 4 shall be used. Based on EN 350-1 [12], the durability rating of Class 3 is classified as moderately durable while Class 4 is slightly durable.

In recent years, due to the diminishing natural timber supply, oil palm trunk (OPT) has been used as an alternative material in wood-based industries, particularly the plywood industry in Malaysia [13]. Based on 100,000 ha of replanting per year, the annual availability of oil palm trunks (OPT) is estimated to be around 13.6 million logs [14]. The total logs available can be converted into 4.5 million cubic meters of plywood under specific controlled processing conditions to manufacture oil palm plywood. However, oil palm plywood is currently used for non-structural materials only, such as cabinets and packaging materials due to some of its imperfections [14].

Oil palm trunks are very hygroscopic in nature and have a very high shrinking and swelling compared to that of wood. The centre part of the oil palm trunk contains only soft parenchyma cells and is not suitable to be used in plywood production. Only the outer part of the trunk can be used in plywood production economically [15]. Moreover, the density of the oil palm trunk varies widely between 200 and 700 kg/m^3^, and its moisture content ranges from 100–500% [16]. These attributes pose a big challenge for the utilization of oil palm trunks in wood-based industries, particularly the saw milling and panel industries. Oil palm plywood has lower strength properties than that of hardwood plywood due to the inferior characteristics of oil palm itself. In addition to their very high moisture content, oil palm trunk contains a high amount of sugar and starch, making it very susceptible to many biodeterioration agents such as fungi and termites [17,18,19]. According to Bakar et al. [20], oil palm wood falls into timber durability Class 5, which is not durable. In the study, oil palm wood was found heavily attacked by subterranean termites and white rot fungi. Due to these shortcomings, oil palm veneers are not suitable to be used to produce marine plywood as timber species of durability rating of at least Class 4 and better shall be used.

Therefore, to eliminate the setbacks posed by the inherited properties of oil palm trunks, the chemical treatment of veneers with phenolic or equivalent resin polymer or other chemicals is required [21]. It is anticipated that the loosely bound parenchyma tissues will significantly absorb the chemicals, and once cured, increase their density. As a result, the density gradient within the veneer is reduced and thus, a stronger and more stable plywood can be produced [22]. Hence, in this study, oil palm trunk veneers were treated with phenol formaldehyde resin prior to consolidation into plywood. An impregnation technique is employed to ensure better polymer retention in the veneers, which then reflects in the performance of the consolidated plywood. The gist of the treatment is that the treated veneers do not require additional glue spreading before being subjected to hot-pressing. A new term, marine grade equivalent (MGE), is coined for the newly produced plywood.

## 2. Materials and Methods

Rotary cut oil palm trunk (OPT) veneers with dimensions of 150 mm long × 150 mm wide × 3–4 mm thick were collected from a local plywood manufacturer located at Batu Kikir, Negeri Sembilan, Malaysia. Medium molecular weight phenol formaldehyde (MmwPF) resin was purchased from Aica Malaysia Sdn. Bhd., Senawang, Selangor, Malaysia. The MmwPF resin has 45% solid content, a viscosity of 50 centipoise, and a molecular weight of around 1500. OPT veneers were treated with MmwPF resin at four different solid contents, namely 15, 20, 30, and 40%, using a simple vacuum method. The MmwPF resin was diluted to 15, 20, 30, and 40% solid content with distilled water using the following equation:

M_1_V_1_ = M_2_V_2_ (1)

where

M_1_ = Solid content of resin PF before dilution (%);

V_1_ = Volume of distilled water that needs to be added (mL);

M_2_ = Target solid content of PF resin after dilution (%);

V_2_ = Volume of resin needed (mL).

The OPT veneers were placed in a treatment cylinder (custom made, Serdang, Selangor, Malaysia) and then a vacuum was applied for 15 min. Then, the cylinder was filled with MmwPF resin, and the OPT veneers were soaked at atmospheric pressure for 5, 15, and 30 min, respectively. After the designated soaking time, the vacuum was applied once again to remove the excessive resin. The treated veneers were then pre-cured in an oven at 70 °C for 10 min.

### 2.1. Treatability of Oil Palm Veneers

Weight percent gain (WPG) and polymer retention (PR) were determined in order to assess the treatability of OPT veneers. After the impregnation treatment, the OPT veneers were assessed for WPG and PR. OPT veneers before and after treatment were weighed and WPG and PR were calculated based on the following equations:

WPG (%): [(W_f_ − W_i_)/W_i_] × 100 (2)

where

WPG = Weight percent gain (%);

W_f_ = Oven dry weight of the OPT veneer after treatment (g);

W_i_ = Initial weight of the OPT veneer before treatment (g).

PR (%): [(W_f_ − W_i_)/W_i_] × C (3)

where

C = Solid content of the MmwPF resin (%);

W_f_ = Weight of OPT veneer after impregnation (g);

W_i_ = Weight of OPT veneer before impregnation (g).

### 2.2. Production of Marine Grade Equivalent (MGE) Plywood

Treated OPT veneers were assembled into 3 layers and hot-pressed in a hot press (Carver CMG 100H-15, Ontario, NY, USA). The treated OPT veneers were assembled into 3-ply and then wrapped in Teflon paper and placed onto a platen plate. The assembly was then pressed into 3-ply MGE plywood using a hot press. Pressing temperatures of 125, 130, 140, and 150 °C and pressing times of 4, 6, 8, 10, 12, 14, and 16 min were attempted to make sure plywood with good bonding was produced.

### 2.3. Dimensional Stability of MGE Plywood

Water absorption (WA) and thickness swelling (TS) of the plywood samples were evaluated. A total of 5 plywood samples with a size of 20 mm × 20 mm × 12 mm were cut from MGE plywood and oven-dried at 103 ± 2 °C. The oven-dried weight and thickness of the MGE plywood were recorded. The dried samples were then subjected to a 30 min vacuum, followed by soaking in distilled water for 24 h. After soaking, the weight and thickness of the samples were measured again and the changes in weight and thickness were expressed in percentage.

### 2.4. Mechanical Performance of MGE Plywood

After conditioning, the MGE plywood was tested for modulus of rupture (MOR) and modulus of elasticity (MOE) according to the British Standard BS 373: 1957 [23] using a Universal Testing Machine (UTM, Instron-3366, Norwood, MA, USA) with modification size of the specimen. For bending, 150 mm × 20 mm × 12 mm specimens were carried out using the centre loading method at a span of 120 mm with a constant crosshead speed of 6.64 mm/min. Load deflection curves were recorded and the values were used to determine MOR and MOE. The equations below were used to calculate MOR and MOE, respectively.

MOR (N/mm^2^) = Pm L/bd^2^ (4)

MOE (N/mm^2^) = P_L_ L^3^/4bd^3^ σ (5)

where

Pm = Maximum load (N);

L = Length of span (mm);

B = Width of the specimen (mm);

d = Thickness of specimen (mm);

P_L_ = Load at proportional limit (N);

Σ = Deflection at mid length at limit of proportionally (mm).

For each bonding quality class, both the mean shear strength and the average apparent cohesive wood failure were determined in accordance with ISO 12466-1 [24]. The MGE plywood shall be pre-treated as specified for the applicable bonding class. For a 24 h cold soak, the MGE plywood was immersed for 24 h in water at a temperature not lower than 17 °C. For a 6 h boil, the plywood sample was boiled for 6 h in boiling water, followed by cooling in water at less than 30 °C for at least 1 h. For the boil-dry-boil (BDB) test, the MGE plywood sample was immersed for 4 h in boiling water, then dried in the ventilated drying oven for 16 h to 20 h at 60 ± 3 °C, then immersed in boiling water for 4 h, followed by cooling in water at less than 30 °C for at least 1 h. The shear test pieces were arranged in the centre of the clamping devices in such a way that the load could be transmitted from the testing machine, via the ends of the shear area without any transverse load. The load was applied at a constant speed to assure the failure occurs within 30 ± 10 s. The load at failure was determined and recorded to an accuracy of 1%. The shear strength was calculated in megapascals. The apparent cohesive wood failure was also determined after the shear test.

### 2.5. Decay Resistance Test

The decay resistance of vacuum impregnated plywood was assessed following the AWPA E10-16 [25]. Test blocks of 25 × 25 × (8, 9, and 10) mm were cut from pre-treated plywood samples. Untreated rubberwood test blocks served as control and reference species in this study. Reference species shall be timber species representative of the country or region of the proposed end-use. In Malaysia, rubberwood has been used as the control for wood treatment studies since it is not naturally durable. Each test block was oven-dried for 2 to 3 days at 103 ± 2 °C to achieve a constant weight, $W_{i}$, which was the initial weight before the decay test. All test blocks were individually wrapped in aluminum foil and steam sterilized for 20 min at 121 °C.

*Pycnoporus sanguineus* white rot fungi, were used as test fungi. *P. sanguineus* was chosen as the test fungus since it is commonly isolated in Malaysia.

Each culture bottle was filled with 150 g of culture soil and 70 mL of distilled water, followed by the placement of a feeder strip on top of the soil in the bottle. Rubberwood feeder strips with the dimension of 3 by 28 by 34 mm were used. All the bottles that were loosely closed with plastic caps were steam sterilized at 121 °C for 20 min and then cooled in a laminar flow. After cooling, an agar disc cut from the edge of an actively growing culture of test fungi was placed on both edges of the feeder strip, and then the culture bottles were incubated in the incubator set at 25 ± 3 °C for at least 2 to 3 weeks until the feeder strips were completely covered by the mycelium.

A test block was placed on top of the mycelium covered feeder strip. The bottles were incubated in a conditioned environment at 25 ± 3 °C for 8 weeks. Then, the test blocks were removed from the bottles at the end of the exposure period and all the mycelium attached to the surface of the blocks was brushed off carefully. After that, the test blocks were placed in an oven at 60 °C for at least two days to achieve constant weight. The weight of the test block after exposure was weighed and recorded, $W_{f}$. The following equation was used to calculate the percent weight loss of each tested block:

(6) $$\mathrm{Weight loss}\;\left(\%\right)=\frac{W_{i}-W_{f}}{W_{i}}\times 100$$

where $W_{i}$ is the weight of the sample before it was exposed to fungi and $W_{f}$ is the weight of the sample after it was exposed to fungi.

### 2.6. Termite Resistance Test

The single-choice test approach was used to evaluate the resistance of treated samples against subterranean termites (*Coptotermes* sp.) in accordance with the AWPA E1-17 Standard [26]. Rubberwood was used as a reference species as it has been used as control for wood treatment studies since it is not naturally durable. The test blocks of 25 × 25 × (8, 9, and 10) mm were cut from vacuum impregnated plywood samples. Untreated samples were used as a control. Prior to testing, the oven-dried test blocks were weighed and recorded, $W_{i}$. Then, the test blocks were placed in test bottles containing 200 g of sand and 30 mL of distilled water. All bottles that were loosely closed with plastic caps were steam sterilized at 121 °C for 20 min and then cooled in a laminar flow. The test block was placed on the sand and without contact with the wall of the culture bottle. In each of the test bottles, 150 termites were introduced, with 15 soldiers and 135 workers. For four weeks, the bottles were covered and incubated in the dark at room temperature. The test blocks were taken out of the bottles at the end of the test to be examined and visually graded.

After oven drying, the weight of the test blocks was also measured and recorded, $W_{f}$. Resistance of termite attack was calculated based on the percentage of weight loss from the equation given as follows.

(7) $$\mathrm{Weight loss}\;\left(\%\right)=\frac{W_{i}-W_{f}}{W_{i}}\times 100$$

where $W_{i}$, is the initial weight of the test block before exposure to termites and $W_{f}$ is the weight of the test block after exposure to termites.

## 3. Results and Discussion

### 3.1. Weight Percent Gain (WPG) and Polymer Retention (PR) of OPT Veneers

**Figure 1:**
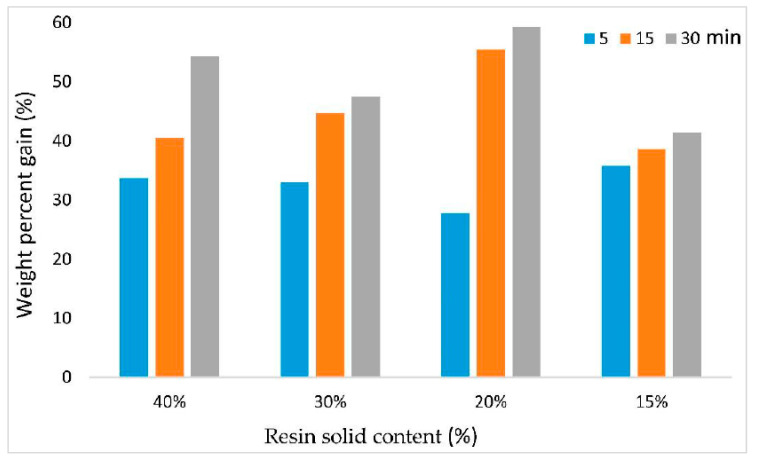
Weight percent gain (WPG) of OPT veneers as a function of resin solid content and soaking time.

**Figure 2:**
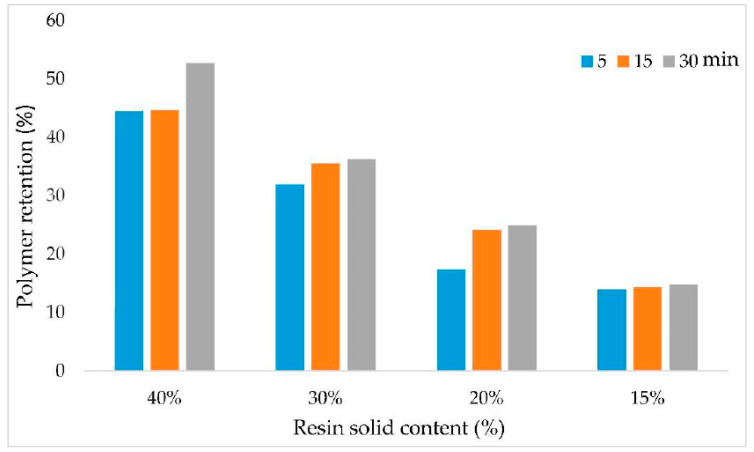
Polymer retention (PR) of OPT veneers as a function of resin solid content and soaking time.

The WPG and PR of OPT veneers as a function of resin solid content and soaking time are shown in Figure 1 and Figure 2, respectively.

The results showed that the WPG of the OPT veneers increased along with the increasing soaking time. Resin solid content exerts an inconsistent effect on the WPG of OPT veneers. OPT veneers treated with 15% PF have the lowest WPG while OPT veneers treated with 20% have the highest WPG, even higher than those of OPT veneers treated with 30% PF. As for PR, PR of the OPT veneers increased along with the increasing soaking time as well as resin solid content. Purba et al. [27] discovered a positive relationship between soaking time with WPG and PR in their study. The authors also stated that the PF resin’s molecular weight and concentration have an impact on the WPG and PR of the treated veneers. The findings were also in agreement with Wang and Zhao [28] who reported that the WPG of the poplar and Chinese fir samples increased as the resin solid content increased from 10 to 48%. Although OPT veneers treated with 40% PF attained the highest PR, it is not economical in practice. Meanwhile, 15% PF did not yield an insufficient PR in the OPT veneers. Therefore, in the next stage of the study, PF resin with 20% and 30% solid content was selected for hot-pressing parameters optimization.

### 3.2. Optimized Pressing Temperature and Time for MGE Plywood

**Figure 3:**
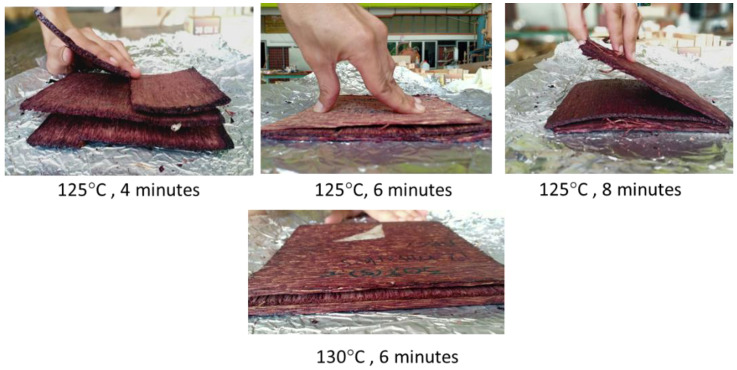
Plywood produced using designated pressing temperatures and times showed failure in bonding.

To obtain an optimum hot-pressing parameter, several attempts were carried out using OPT veneers treated with 30% solid content and pressed under different pressing parameters (temperature and time). Firstly, pressing temperatures of 125 and 130 °C and pressing temperatures of 4, 6, and 8 min were used. However, it was found that these pressing temperatures and pressing times were not suitable for the veneers treated with 30% PF. As shown in Figure 3, plywood produced using these hot-pressing temperatures and times did not fully bond. Delamination occurred right after hot-pressing. Therefore, a higher pressing temperature and longer pressing time were used to improve the bonding of MGE plywood. Pressing temperatures of 140 and 150 °C and pressing times of 8, 10, 12, and 14 min were used. It was found that the workable pressing temperature and time to achieve good bonding are 140 °C or higher temperature for 10 min or longer for bonding OPT veneers treated with 30% PF. According to Li et al. [29], at lower pressing temperatures, the adhesive tends to have lower fluidity, causing the moisture in the core layer of the plywood to not evaporate rapidly. As a result, the steam pressure is concentrated at the core layer and negatively impacted the bonding between plies. Higher pressing temperatures, on the other hand, shortened the rapid heating stage, allowing the adhesive to cure more completely and resulting in better bonding. Li et al. [29] also stated that a longer pressing time improved plywood bonding, which is consistent with the findings of this study.

As a result, the following parameters as shown in Table 1 were used for the production of MGE plywood. The produced plywood was tested for dimensional stability, mechanical performance, decay, and termite resistance.

### 3.3. Dimensional Stability and Mechanical Performance of MGE Plywood

Density, water absorption (WA), and thickness swelling (TS) of MGE plywood produced using different solid content, soaking time, pressing temperature, and time is shown in Table 2. It can be seen that MGE plywood pressed at 150 °C for 12 min has the lowest density. MGE plywood pressed at 140 °C for 12 min and 150 °C for 10 min has a density of 651 kg/m^3^ and 628 kg/m^3^, respectively. Plywood with a lower density tends to retain more water, thus having a higher water absorption (101%) but lower thickness swelling (4.04%). Over-pressing of the samples at higher temperatures and longer pressing times might be the reason that led to such an observation. A pressing time of 12 min at 150 °C might be too severe for the plywood. PF resin in the OPT veneers might be excessively evaporated during hot-pressing and leave behind a porous structure of plywood with the lowest density. Correspondingly, water penetrated easily and resulted in high water absorption. Meanwhile, plywood with higher porosity swells to a smaller extent as the voids could accommodate a higher amount of water.

The shear strength of MGE plywood produced using different solid content, soaking time, pressing temperature, and time is shown in Table 3. MGE plywood pressed at 140 °C for 12 min has the highest shear strength, followed by those pressed at 150 °C for 10 min. Both samples surpassed the requirement stipulated in ISO 12466-2 [30], where the minimum shear strength of the plywood shall be more than 1.0 MPa. In the case of MGE plywood that was pressed at 150 °C for 10 min, the shear strength after boil-dry-boil (BDB) treatment is 0.95 MPa, slightly lower than the requirement of 1.0 MPa. However, it has a cohesive wood failure of 83%. According to ISO 12466-2 [30], each glue line tested shall satisfy two criteria which are the mean shear strength and the average apparent cohesive wood failure. For samples that have a shear strength of 0.6 to 1.0 MPa, cohesive wood failure of more than 40% is required. Therefore, these samples (83% wood failure) have also surpassed the requirement.

Meanwhile, MGE plywood pressed at 150 °C for 12 min has the lowest shear strength of 0.52 MPa. For shear strength falling into this class (0.4 to 0.6 MPa), the samples shall have a cohesive wood failure of more than 60% in order to meet the requirement. Unfortunately, the wood failure of the sample was 50%, thus it failed to meet the requirement stipulated in ISO 12466-2 [30]. The sample failed after 24 h soaking in water as well as after a 6 h boil and BDB treatment. As mentioned earlier, a high pressing temperature and long pressing time might have caused the PF resin to evaporate from the OPT veneers and is insufficient to yield a satisfactory bonding. Another explanation is that longer pressing times at high temperatures caused glue lines to age, resulting in a lower shear strength [29].

Table 4 displays the modulus of rupture (MOR) and modulus of elasticity (MOE) of MGE plywood produced using different solid content, soaking time, pressing temperature, and time. A similar observation was also made as the MGE plywood pressed at 140 °C for 12 min exhibited the highest MOR and MOE.

### 3.4. Decay Resistance of MGE Plywood

**Figure 4:**
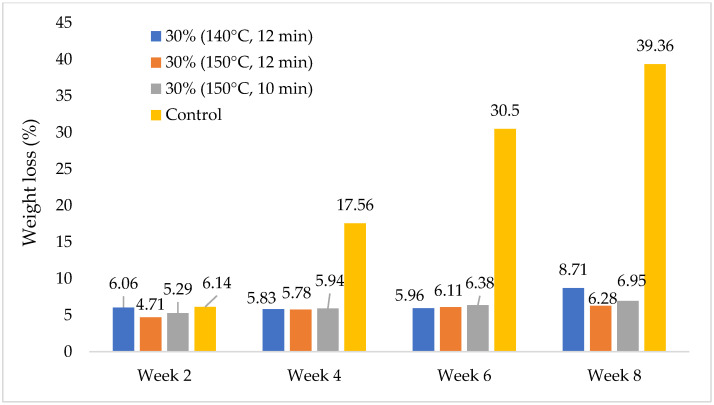
Weight loss of samples after 2-, 4-, 6-, and 8-weeks exposure to white rot fungi.

Decay resistance of the MGE plywood after exposure to white rot fungi is displayed in Figure 4. After a two-week exposure, MGE plywood exhibited a slightly lower weight loss (4.71 to 6.06%) compared to that of the control rubberwood (6.14%). The difference became more obvious after exposure for 4 weeks and above. After 8 weeks of exposure, control rubberwood lost 39.36% of its initial weight while MGE plywood merely lost 6.28 to 8.71%. Among this, MGE plywood pressed at 150 °C for 12 min has the lowest weight loss of 6.28%, indicating the highest resistance against white rot fungi. Control rubberwood samples fell into natural durability of Class 5—non-durable (weight loss of more than 30%)—while MGE plywood has a durability class of Class 2—durable (weight loss of 5–10%) [12]. Improvement in decay resistance is often observed on PF-treated wood [31]. The improvement in decay resistance might be due to the fact that the cell wall of the OPT veneers was diffused by PF resin and, therefore, inhibited the penetration by fungi [32]. Furthermore, PF-treated OPT veneers have lower moisture absorption and diffusivity. As a result, this made it more difficult for white rot fungi to proliferate and survive on the samples [33].

**Figure 5:**
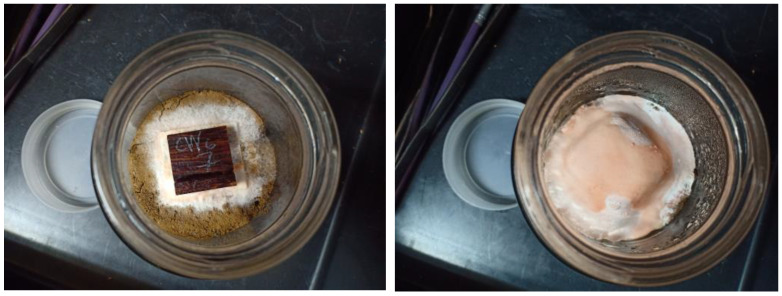
Fungal growth observation of the MGE plywood (**left**) and control (**right**) after 8-week exposure to white rot fungi.

**Figure 6:**
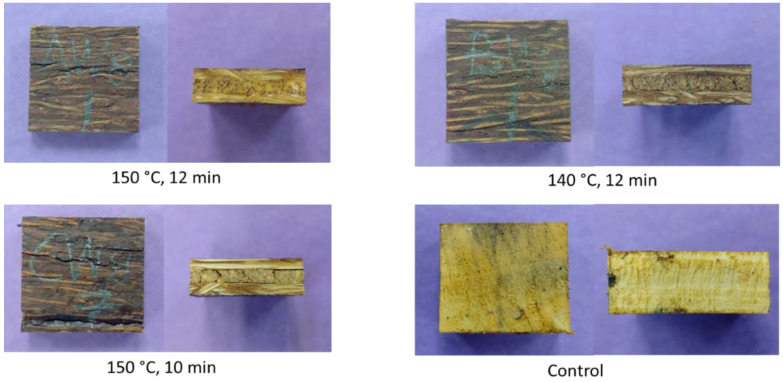
Visual appearance of the MGE plywood and control samples after an 8-week exposure to white rot fungi.

Figure 5 shows the fungal growth observation of the MGE plywood and control samples after 8-week exposure to white rot fungi. As can be seen, the control sample was fully covered by the white rot fungi. On the contrary, no growth was observed on the surface of MGE plywood. The visual appearances of the samples after 8-week exposure are shown in Figure 6. No visible damage caused by white rot fungi was observed on the MGE plywood samples. The cracks on the MGE plywood samples were natural cracking after hot-pressing. Meanwhile, signs of white rot infestations were observed on the surface of rubberwood samples.

### 3.5. Termite Resistance of MGE Plywood

**Figure 7:**
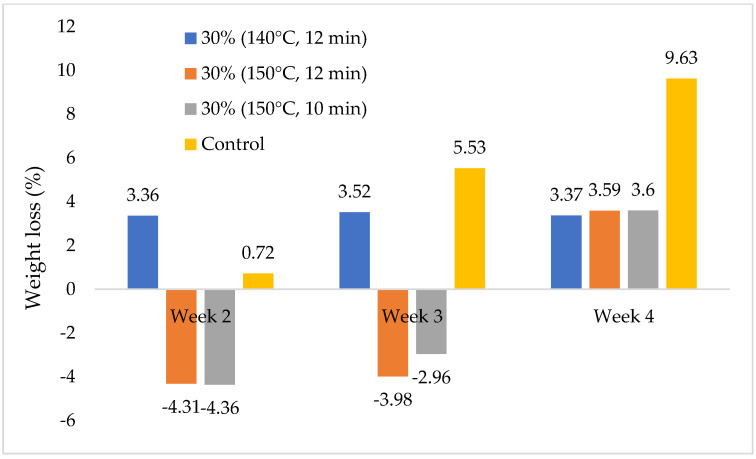
Weight loss of samples after 2-, 3-, and 4-weeks exposure to subterranean termites.

**Figure 8:**
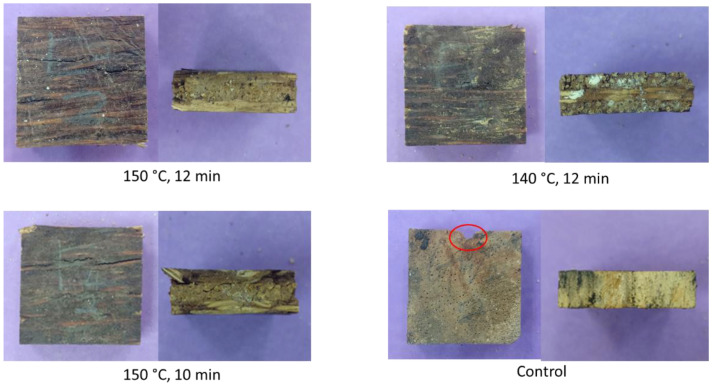
Visual appearance of the MGE plywood and control samples after a 4-week exposure to subterranean termites. Note: Red circle in control sample showed sign of attack by termites.

Termite resistance of the MGE plywood after exposure to subterranean termites are displayed in Figure 7. At week 2 and week 3, the weight loss of the samples was not prominent. MGE plywood even experienced a gain in weight (indicated by a negative weight loss value) due to the water added to the bottles during the test. After a 4-week exposure, it can be clearly seen that the weight loss of control rubberwood samples is almost three-fold to that of the MGE plywood. The visual appearance of the samples after four weeks of termite exposure is shown in Figure 8. MGE plywood exhibited a visual rating score of 10 where the samples are sound and free of traces of attacks by termites. Control rubberwood samples have a visual rating score of 8 where it was moderately attacked by termites with 3–10% of the cross-sectional area affected. According to the wood durability classification against soil termites reported by Trisatya et al. [34], the control rubberwood fell into Class III—moderately durable class (mass loss of 7.50 to 10.96%). MGE plywood pressed at 150 °C for 12 min was classified as Class I—very durable (3.59% mass loss).

It is suspected that termites were unable to digest PF which led to high mortality rates for termites [35]. PF might also be poisonous to termites and result in better resistance of the MGE plywood towards termite attack [20]. Because of the penetration of PF polymer into the OPT intercellular cell wall, a hardened polymer network is formed, which inhibits termite attacks [36]. Termite feeding activity, on the other hand, is highly dependent on the moisture content of the wood [37,38]. As a result of the reduction in moisture absorption caused by the PF impregnation treatment, MGE plywood is less susceptible to termite attacks [39].

## 4. Conclusions

Marine grade equivalent (MGE) plywood has been successfully produced in this study using phenolic resin treated oil palm trunk (OPT) veneers. MGE plywood produced displayed a satisfactory bonding quality and bending strength provided a suitable pressing temperature and time were employed. It is recommended that the lower pressing temperature shall be used in the manufacturing of MGE plywood to avoid over-evaporation of PF resin in the OPT veneers. In terms of decay and termite resistance, MGE plywood exhibited superior resistance against both of these biodeterioration agents. MGE plywood produced in this study achieve a durability rate of Class 1 (very durable) against white rot fungi decay, compared to that of Class 5 (not durable) in reference to rubberwood samples. In terms of termite resistance, MGE plywood has a durability rate of Class II (durable) and Class I (very durable) compared to that of Class III (moderately durable) in reference to rubberwood samples. The results revealed the feasibility of the production of MGE plywood using OPT veneers. Nevertheless, more efficient processing parameters have to be identified and improved in future studies.

## Figures and Tables

**Table 1:** Processing parameters used for the production of MGE plywood in this study.

Parameters	Solid Content (%)	Soaking Time under Pressure (min)	Pressing Temperature (°C)	Pressing Time (min)
A	30	15	140	12
B	30	15	150	12
C	30	15	150	10

**Table 2:** Density, water absorption (WA), and thickness swelling (TS) of MGE plywood produced using different solid content, soaking time, pressing temperature, and time.

Solid Content (%)	Soaking Time (min)	Pressing Temp. (°C)	Pressing Time (min)	Density (kg/m^3^)	Thickness Swelling (%)	Water Absorption (%)
30	15	140	12	651	6.1	57
30	15	150	12	474	4.04	101
30	15	150	10	628	5.04	67

**Table 3:** Shear strength of MGE plywood produced using different solid content, soaking time, pressing temperature, and time.

Solid Content (%)	Soaking Time (min)	Pressing Temp. (°C)	Pressing Time (min)	Dry Shear (MPa)	24 h Cold Soak Shear (MPa)	6 h Boil Shear (MPa)	BDB Shear (MPa)
30	15	140	12	3.71	2.98	2.63	1.55
30	15	150	12	0.52	Sample Failed	-	-
30	15	150	10	2.13	2.34	1.93	0.95

**Table 4:** Modulus of rupture (MOR) and modulus of elasticity (MOE) of MGE plywood produced using different solid content, soaking time, pressing temperature, and time.

Solid Content (%)	Soaking Time (min)	Pressing Temp. (°C)	Pressing Time (min)	MOR (MPa)	MOE (MPa)
30	15	140	12	38.85	5120
30	15	150	12	32.19	4110
30	15	150	10	31.03	4400

## Data Availability

Not applicable.
